# Investigation of the mechanism of action of mefloquine and derivatives against the parasite *Echinococcus multilocularis*

**DOI:** 10.1016/j.ijpddr.2023.03.002

**Published:** 2023-03-09

**Authors:** Roman Memedovski, Matías Preza, Joachim Müller, Tobias Kämpfer, Reto Rufener, Marcus Vinicius Nora de Souza, Emerson Teixeira da Silva, Gabriel Fernandes de Andrade, Sophie Braga, Anne-Christine Uldry, Natasha Buchs, Manfred Heller, Britta Lundström-Stadelmann

**Affiliations:** a Institute of Parasitology, Department of Infectious Diseases and Pathobiology, Vetsuisse Faculty, University of Bern, Bern, Switzerland; b Fundação Oswaldo Cruz, Instituto de Tecnologia em Fármacos – Far Manguinhos, 21041-250, Rio de Janeiro, Brazil; c Proteomics and Mass Spectrometry Core Facility, Department for BioMedical Research (DBMR), University of Bern, Bern, Switzerland; d Multidisciplinary Center for Infectious Diseases, University of Bern, Bern, Switzerland

**Keywords:** *Echinococcus multilocularis*, Alveolar echinococcosis, Mefloquine, Structure-activity relationship (SAR), Mode of action, nLC-MS/MS, Ferritin, Energy metabolism

## Abstract

Alveolar echinococcosis (AE) is caused by infection with the fox tapeworm *E. multilocularis*. The disease affects humans, dogs, captive monkeys, and other mammals, and it is caused by the metacestode stage of the parasite growing invasively in the liver. The current drug treatment is based on non-parasiticidal benzimidazoles. Thus, they are only limitedly curative and can cause severe side effects. Therefore, novel and improved treatment options for AE are needed.

Mefloquine (MEF), an antimalarial agent, was previously shown to be effective against *E. multilocularis in vitro* and in experimentally infected mice. However, MEF is not parasiticidal and needs improvement for successful treatment of patients, and it can induce strong neuropsychiatric side-effects. In this study, the structure-activity relationship and mode of action of MEF was investigated by comparative analysis of 14 MEF derivatives. None of them showed higher activity against *E. multilocularis* metacestodes compared to MEF, but four compounds caused limited damage. In order to identify molecular targets of MEF and effective derivatives, differential affinity chromatography combined with mass spectrometry was performed with two effective compounds (MEF, MEF-3) and two ineffective compounds (MEF-13, MEF-22). 1′681 proteins were identified that bound specifically to MEF or derivatives. 216 proteins were identified as binding only to MEF and MEF-3. GO term enrichment analysis of these proteins and functional grouping of the 25 most abundant MEF and MEF-3 specific binding proteins revealed the key processes energy metabolism and cellular transport and structure, as well as stress responses and nucleic acid binding to be involved. The previously described ferritin was confirmed as an exclusively MEF-binding protein that could be relevant for its efficacy against *E. multilocularis*. The here identified potential targets of MEF will be further investigated in the future for a clear understanding of the pleiotropic effects of MEF, and improved therapeutic options against AE.

## Highlights

• *E. multilocularis* metacestodes cause alveolar echinococcosis (AE) in humans.
• Current AE treatment is based on benzimidazoles with parasitostatic effects.
• Mefloquine (MEF) acts *in vitro* and in mice against metacestodes.
• MEF derivatives for analysis of structure activities and modes of action.
• Energy metabolism and cellular transport as MEF-binding target pathways.

## Graphical abstract

**Figure:**
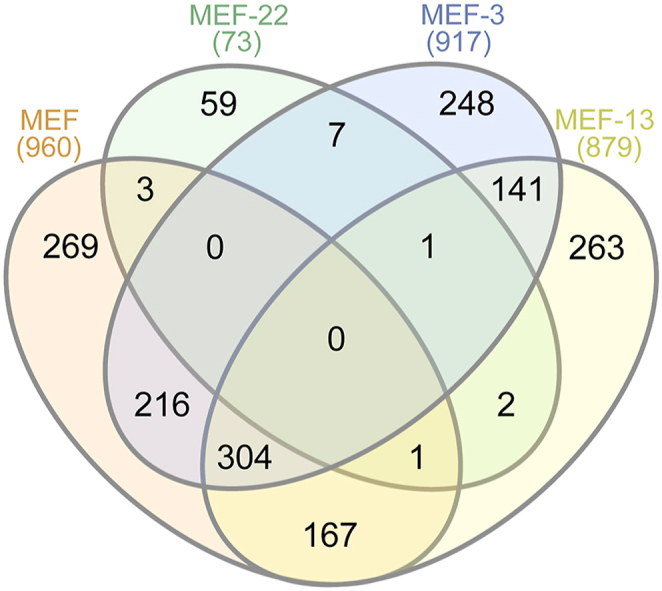

## 1 Introduction

The larval metacestode stage of the small fox tapeworm *Echinococcus multilocularis* causes the disease alveolar echinococcosis (AE). The zoonosis involves carnivores as definitive hosts and a diversity of small mammalian species (mostly rodents) as intermediate hosts. Humans, monkeys and other mammals can be affected as aberrant hosts when infected with eggs from *E. multilocularis* (Lymbery et al., 2017). The resulting disease AE is characterized by the clustered, tumor-like growth of metacestodes mainly in the liver of patients. The course of the disease is asymptomatic in the first years of infection, but when untreated, AE becomes progressive and life-threatening. *E. multilocularis* ranks first and third in the respective European and global rankings of food-borne parasites (Bouwknegt et al., 2018). This is due to the fact that AE is fatal in untreated patients, and curative drug treatment lacks to date (Lundström-Stadelmann et al., 2020). Treatment options rely on complete surgical resections performed in 20–50% of infected people in countries where healthcare infrastructure is easily accessible (Kern et al., 2017). Following surgery, patients are recommended for chemotherapeutic treatment with the benzimidazoles (BMZs) albendazole or mebendazole for at least two years, and for regular monitoring for at least ten years (Brunetti et al., 2010). Unfortunately, AE is usually diagnosed at a late, already advanced stage, making radical surgical treatment difficult or impossible. Then the only treatment option left is based on BMZ treatment. Chemotherapeutic treatment with BMZ is limited by parasitostatic effects and therefore requires long-term (often life-long) treatment (Lundström-Stadelmann et al., 2020). BMZ are tolerated in 70–80% of AE patients, with elevated transaminases, proteinuria, transient hair loss, gastrointestinal disturbances, leukopenia and neurological symptoms as the most common adverse effects (Kern et al., 2017). In a German long-term observational study, 6.9% suffered from severe liver toxicity (Grüner et al., 2017). Thus, there is an urgent need for new drugs to successfully treat AE.

In a drug-repurposing approach, the anti-malarial mefloquine (MEF) was used against *E. multilocularis in vitro* and in infected mice and in these studies MEF exhibited promising, albeit not parasiticidal, activity (Küster et al., 2011, 2015; Lundström-Stadelmann et al., 2020). When used in malaria prophylaxis, mefloquine can induce strong neuropsychiatric side-effects (Tickell-Painter et al., 2017). A first clinical report of an AE patient that was treated with MEF did not lead to a positive treatment response, but no adverse effects did occur either (Burkert et al., 2022). This further confirms the potential lack of parasiticidal activity of MEF in an *in vivo* setting. Comparative *in vitro* screening of MEF derivatives was performed against *E. multilocularis* metacestodes, but none of the tested compounds showed improved activity compared with MEF (Rufener et al., 2018).

Though several targets are described for MEF in *Plasmodium*, bacteria and mammals (Ghosh et al., 2021), the molecular targets of MEF in *E. multilocularis* are unknown. Former investigations using affinity chromatography revealed enolase as a major MEF-binding protein in *Schistosoma mansoni* (Manneck et al., 2012), ferritin in *E. multilocularis* (Küster et al., 2015), and nicotinamide phosphoribosyltransferase in human cells (Küster et al., 2015). In these studies, the major binding proteins were identified after SDS-PAGE of eluates followed by staining and excision of the most visible bands, which may have caused a bias. Moreover, appropriate controls, i.e. pull-downs with ineffective MEF derivatives, were lacking. Therefore, in order to get up-to-date insight into molecular targets of MEF, another methodology was used in this study. Differential affinity chromatography (DAC) is based on pull-downs of cell free extracts on columns coated with effective compounds or ineffective derivatives followed by nano-liquid chromatography coupled to tandem mass spectrometry (nLC-MS/MS) of the eluates. In recent studies, DAC has been used to identify molecular targets of ruthenium complexes in *Toxoplasma gondii* and *Trypanosoma brucei* (Anghel et al., 2021), and to identify molecular targets of a bumped kinase inhibitor in *Neospora caninum* and *Danio rerio* (Müller et al., 2022a) and proteins binding to Leucinostatin-like peptides (Müller et al., 2022b).

Taking advantage of the investigation of a new series of MEF derivatives followed by a structure activity relationship (SAR) study, we performed DAC with effective and ineffective MEF derivatives in order to identify potential drug targets of MEF in *E. multilocularis* metacestodes.

## 2 Materials and methods

All chemicals were purchased from Merck (Darmstadt, Germany), if not stated otherwise. Cell culture reagents were from Gibco (through Fisher Scientific AG, Reinach, Switzerland) if not indicated differently.

### 2.1 Synthesis and chemical analysis of 14 MEF derivatives

**Fig. 1:**
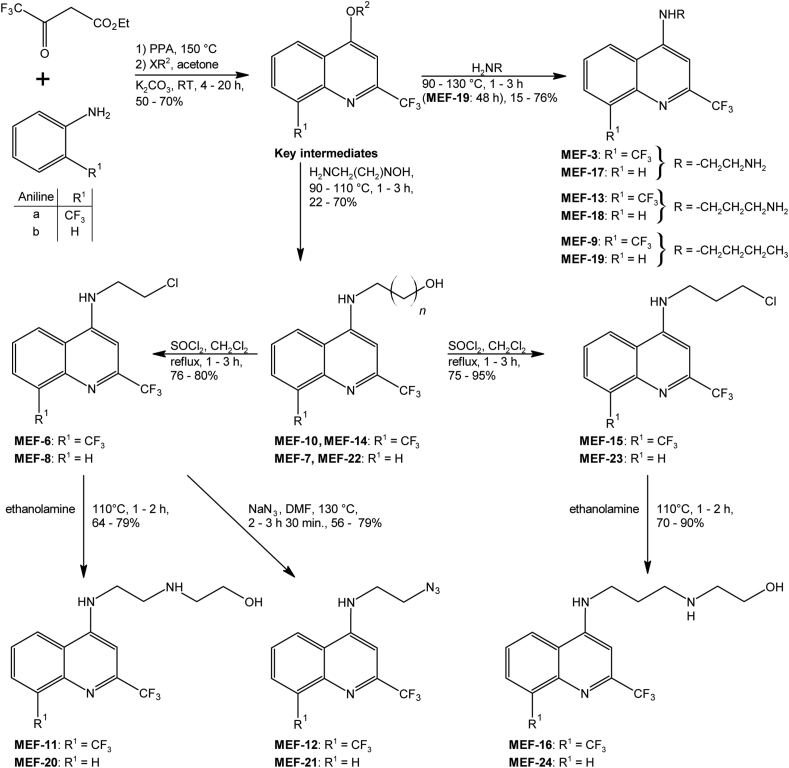
**Synthetic route for the generation of substituted 4-N-alkylated-2-trifluoromethylquinoline analogues.** The quinoline core structure with free hydroxyl groups (called key intermediates) is the precursor of the various MEF derivatives and is synthesized from substituted anilines (da Silva et al., 2021).

The structures of MEF derivatives are given in Fig. 1. The MEF derivatives (Fig. 1) were synthesized at Fundação Oswaldo Cruz as previously described (da Silva et al., 2021). MEF-1 to MEF-10 were previously applied in Rufener et al. (2018). MEF-11 to MEF-24 had not previously been tested against *E. multilocularis* and were tested in this study. In short, the first step was the condensation of substituted anilines with ethyl 4,4,4-trifluoro-3-oxobutanoate in presence of PPA at 150 °C for 2–3 h to induce formation of the substituted quinoline nucleus with a free hydroxyl group at position 4. These phenols were then alkylated with methyl iodide or ethyl bromide in acetone at room temperature in the presence of Na_2_CO_3_ for 4–20 h to produce the key intermediates with a 50–70% yield, respectively. These alkylated compounds were subjected to nucleophilic substitution with diamines, alkylamine, and amino alcohols at 90–120 °C to produce the derivatives MEF-3, MEF-7, MEF-9, MEF-10, MEF-13, MEF-14, MEF-17, MEF-18, MEF-19, and MEF-22 with a 15–76% yield after 1–3 h of reaction (MEF-19: 48 h). The compounds MEF-7, MEF-10 and MEF-14, MEF-22 were converted to the chloro-derivatives MEF-6, MEF-8 and MEF-15, MEF-23 by treatment with SOCl_2_ in CH_2_Cl_2_ or CHCl_3_ at reflux with a 75–95% yield after 1–3 h of reaction. The compounds MEF-11, MEF-20 were obtained after reaction of MEF-6, MEF-8 with ethanolamine under heating at 110 °C for 1–2 h with a 64–79% yield. The azido compounds MEF-12, MEF-21 were obtained after reaction of MEF-6, MEF-8 with sodium azide in DMF at 130 °C for 2–3:30 h in 56–79% yield. The compounds MEF-16, MEF-24 were obtained after reaction of MEF-15, MEF-23 with ethanolamine at 110 °C for 1–2 h in 70–90% yield.

All these compounds were identified by detailed spectral data, including ^1^H-NMR, ^13^C-NMR, and high resolution mass spectra. In general, the ^1^H-NMR spectrum showed four or five quinoline protons at 8.90–6.20 ppm and the aliphatic protons at 4.00–0.95 ppm. The ^13^C-NMR spectrum showed the quinoline carbon signals at the region of 93–165 ppm and the aliphatic carbons at the regions of 14–61 ppm. The CF_3_ group showed a quartet with J about 270–274 Hz. In the infrared spectra, characteristic signals of NH were observed at 3200 - 3380 cm^−1^, OH signals were observed at 3500 - 4000 cm^−1^ and N_3_ signals were observed at 2090 - 2100 cm^−1^. For all compounds the C–F axial deformation signals were observed at 1080 - 1300 cm^−1^.

Melting points were determined with a MQAPF-302 Micro Química apparatus and are uncorrected. NMR spectra were determined using 400 or 500 MHz Bruker AC spectrometers using tetramethylsylane as internal standard. Splitting patterns are as follows: s, singlet; d, duplet; d, double duplet; t, triplet; quin, quintet; m, multiplet; Brl, broad signal. Infrared spectra were obtained using a Thermo Nicolet 6700 spectrometer. Mass spectra were recorded on Agilent 122 5532 GC/MS column by electron impact and high resolution spectra on Bruker compact-Tof. The progress of the reactions was monitored by thin-layer chromatography (TLC) on 2.0 cm × 6.0 cm aluminium sheets (silica gel 60, HF-254, Merck) with a thickness of 0.25 mm, ultraviolet light irradiation. For column chromatography, Merck silica gel (70–230 or 230–400 mesh) was used. Solvents and reagents were used without further purification.

### 2.2 *E. multilocularis* strain maintenance in mice

Female BALB/c mice (Charles River, Sulzfeld, Germany) were used for parasite strain maintenance at the Institute of Parasitology in Bern. They had access to food and water *ad libitum* and were kept in ventilated cages with a controlled temperature of 21 °C–23 °C, a relative humidity of 45%–55%, and a 12/12 h light/dark cycle. Animals were kept under the cantonal license number BE30/19 approved by the Animal Welfare committee of the canton of Bern. All animals were treated in compliance with the Swiss Federal Protection of Animals Act (TSchV, SR455). Intraperitoneally infected BALB/c mice were euthanized after 2–4 months post infection to isolate *E. multilocularis* metacestodes (isolate H95) as previously described (Rufener et al., 2018).

### 2.3 *E. multilocularis* metacestode *in vitro* culture

*E. multilocularis* metacestodes were cultured as described before at the Institute of Parasitology Bern (Rufener et al., 2018). The metacestode material resected from infected mice was incubated overnight at 4 °C with 100 U/ml penicillin, 100 μg/ml streptomycin, 10 μg/ml tetracycline and 20 μg/ml levofloxacin. The parasite pellet was taken up in Dulbecco's Modified Eagle Medium (DMEM) with phenol red, 4.5 g/l D-glucose and pyruvate, supplemented with 10% fetal calf serum (FCS, Biochrom GmbH, Berlin, Germany), 100 U/ml penicillin, 100 μg/ml streptomycin and 10 μg/ml tetracycline. The suspension was transferred to a 75 cm^2^ (T75) culture flask with confluent Reuber rat hepatoma (RH) feeder cells. The new co-culture was incubated at 37 °C and 5% CO_2_ under humid atmosphere. The medium of the co-culture was changed once a week. The RH culture was maintained in the same culture medium and the same conditions as the metacestodes and passaged once a week to be added to the metacestodes.

### 2.4 *In vitro* drug screening against *E. multilocularis* metacestodes

14 MEF derivatives (MEF-11 to MEF-24) were tested against *E. multilocularis* metacestodes by PGI assay, which measures damage of metacestodes via the damage marker phosphoglucose isomerase (PGI) (Stadelmann et al., 2010). Metacestodes of 6–8 weeks of age were used for these *in vitro* tests. Purified metacestodes were distributed to 48 well plates diluted 1:2 in DMEM (without phenol red, supplemented with 100 U/ml penicillin, 100 μg/ml streptomycin). In a first screen, all 14 MEF derivatives were tested in triplicates at a final concentration of 40 μM in 0.1% dimethyl sulphoxide (DMSO), with an incubation time of 5 days, 5% CO_2_, humid atmosphere. In a second screen, the five most effective MEF derivatives (>20% activity in first screen) were tested at 40 μM, 30 μM, 20 μM and 10 μM, each in triplicate, for 5 days, 5% CO_2_, humid atmosphere. For all assays, respective DMSO (0.1%) and Tx-100 (0.1%) controls were included in triplicates. After 5 days of metacestode incubation, supernatants were collected and stored at – 20 °C until measurement. PGI activities were measured indirectly via formation of NADH as described before (Stadelmann et al., 2010). Activities were calculated as percentage of the Tx-100 control. They are given as mean values and standard deviations for each triplicate.

### 2.5 Affinity chromatography of *E. multilocularis* metacestode proteins to MEF derivatives

(a) Coupling of MEF-ligands to Sepharose matrix

The coupling of MEF, MEF-3, MEF-13 and MEF-22 to epoxy-activated sepharose® 6B with a C12 spacer arm was performed according to the manufacturer's instructions as described previously (Müller et al., 2008) with the exception that MEF or derivatives were used as ligands and not thiazolides. In short, 0.5 g of lyophilized epoxy-sepharose® 6B (Sigma-Aldrich, St. Louis, Mo, USA) was suspended in 15 ml H_2_O (Bichsel AG, Interlaken, Switzerland) and centrifuged at 300×*g* for 5 min at 4 °C. Washing in water was repeated twice, followed by a two-stage wash with coupling buffer (0.1 M Na_2_CO_3_, pH 9.5). 15.5 ± 0.3 mg of the ligand was weighed and dissolved in 1 ml DMSO, added to 1.5 ml of the washed epoxy-sepharose resin and incubated for 96 h at 37 °C on a horizontal shaker with slow but continuous shaking (150 rpm) to allow the drug to react in a nucleophilic addition to the epoxy-activated sepharose® 6B. After 72 h of incubation, 2 ml of DMSO was added to the resin to dissolve all excess and possibly precipitated drug in the supernatant to further increase the coupling efficiency. After incubation, the matrix was washed again with coupling buffer, followed by another wash with ethanolamine (1 M, pH 9.5). The matrix was overlaid with ethanolamine (1 M, pH 9.5) and incubated for 4 h at 20 °C on a horizontal shaker at 150 rpm in the dark to block residual groups. The resulting column medium was then transferred to an empty PD-10 column (GE Healthcare, Glattbrugg, Switzerland) and washed with PBS/DMSO (1:1) (5 column volumes) to remove unbound ligands. The resulting column was stored sealed in PBS containing 0.02% NaN_3_ at 4 °C until use. Mock columns were prepared in the same way, but without any ligand.

(b) Metacestode protein extraction for affinity chromatography

Extracts were prepared from *in vitro* cultured *E. multilocularis* metacestodes maintained under axenic aerobic conditions (5% CO_2_, 20% O_2_, humid atmosphere, 37 °C) without RH cells or FCS and DMEM containing 150 U/ml penicillin and 150 μg/ml streptomycin. After 72 h, the metacestodes were washed extensively in PBS, destroyed after removal of all PBS and centrifuged at 500×*g* for 5 min at 4 °C. This washing step was performed twice more. To lyse the cells in the tissue pellet, 4 ml of PBS containing Tx-100 (0.1%), 5 mM EDTA and 10 μl/ml Halt Protease Inhibitor cocktail (Thermo Fisher) were added and then vortexed for 1 min. After centrifugation of the suspension at 4000×*g* for 10 min, 4 °C, 8 ml of supernatant was re-centrifuged in 1.5 ml Eppendorf tubes at 12′000×*g* for 10 min at 4 °C. The supernatant corresponding to the cell extract was immediately used for differential affinity chromatography.

(c) Differential affinity chromatography (DAC)

Differential affinity chromatography was performed similarly as described previously (Anghel et al., 2021; Müller et al., 2022a, 2022b). Parasite extracts were passed first through a mock column (Küster et al., 2015) followed by a column containing effective or ineffective drugs and at a flow rate of 0.25 ml/min. Prior to elution, the columns were washed with at least 5 column volumes of PBS. Mock and drug columns were eluted with 4 ml of acetic acid (50 mM, pH 2.9) each. The eluates were aliquoted (1 ml) and lyophilized overnight on a Savant SC100 SpeedVac concentrator for subsequent analysis by nano-liquid chromatography coupled tandem mass spectrometry (nLC-MS/MS) as described previously (Müller et al., 2022b).

### 2.6 Mass spectrometry and data processing

nLC-MS/MS was performed to identify the protein composition of the above-described samples (a) and a specific workflow of data processing and visualization (b), protein annotation (c) and Gene Ontology enrichment analysis (d) was used.

(a) nLC-MS/MS

At the Proteomics and Mass Spectrometry Core Facility, University of Bern, eluates were re-suspended in 10 μl lysis buffer (8 M Urea/100 mM Tris-HCl pH8), reduced by addition of 1 μl 0.1 M DTT for 30 min at 37 °C, alkylated with 1 μl of 0.5 M iodoacetamide for 30 min at 37 °C in the dark, followed by quenching of iodoacetamide by the addition of 5 μl 0.1 M DTT and digested with LysC for 2 h at 37 °C followed by Trypsin overnight at room temperature. The digests were analysed by liquid chromatography on an Ultimate 3000 (Thermo Fisher, Bremen, Germany) coupled to a LUMOS tribrid orbitrap mass spectrometer (Thermo Fisher, San José, USA) with two injections of 500 ng peptides. The samples were loaded in random order onto a pre-column (C18 PepMap 100, 5 μm, 100 A, 300 μm i.d. X 5 mm length) at a flow rate of 10 μl/min with solvent C (0.05% TFA in water/acetonitrile 98:2). After loading, peptides were eluted in back flush mode onto a homemade C18 CSH Waters column (1.7 μm, 130 Å, 75 μm × 20 cm) by applying a 90-min gradient of 5% acetonitrile to 40% in water, 0.1% formic acid, at a flow rate of 250 nl/min.

Data acquisition was made in data dependent mode with precursor ion scans recorded in the orbitrap at a resolution of 120′000 (at m/z = 250) parallel to top speed fragment spectra of the most intense precursor ions in the linear iontrap for a cycle time of 3 s maximum, applying a 30-s exclusion time window.

(b) Data processing and visualization

Mass spectrometry data was processed by MaxQuant software (version 1.6.14.0) using default parameter settings with 10 ppm mass accuracy for precursor ion masses in the first search run, and 0.4 Da mass accuracy for fragment spectra recorded in the linear iontrap. A fixed modification of carbamidomethylation on cysteines and variable modifications of protein N-terminal acetylation, oxidation on methionine, and deamidation of asparagine and glutamine were set for a database search against echinococcus_multilocularis.PRJEB122.WBPS15.protein_1.fasta (download date March 9, 2021) sequence database deactivation match between runs option. Proteins at a 1% FDR with at least two razor and unique peptide identifications (also with a 1% FDR setting) were accepted as present in the sample.

**Fig. 2:**
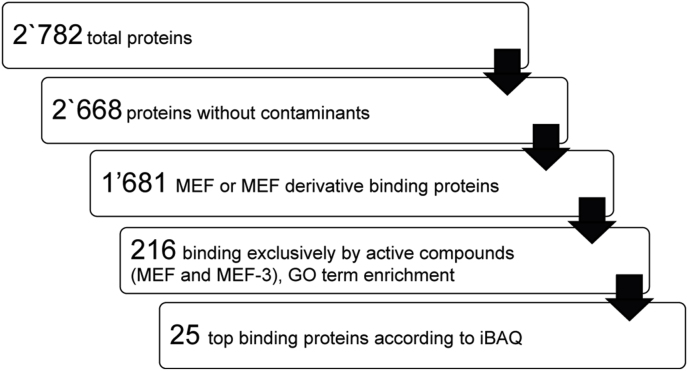
**Data processing workflow for analysis of the *E. multilocularis* metacestode proteome acquired by nLC-MS/MS after DAC with MEF, MEF-3, MEF-13 and MEF-22.** From the total proteins, contaminants and proteins binding to the mock columns were subtracted. GO enrichment was performed for proteins binding only to the active compounds MEF and MEF-3.

Subsequent data analysis was performed in Microsoft Excel 2019, applying the data processing workflow as follows (Fig. 2): Only strictly detected lead proteins in both duplicates per drug column and not in the corresponding mock column were characterized. To reduce the number of non-specifically binding proteins, only those proteins that were bound exclusively by the effective drugs MEF and MEF-3 were considered. Finally, hits were sorted by descending iBAQ values, which are the quotients of their intensities and the number of tryptic peptides, to determine the top 25 bound proteins by MEF and MEF-3. For Venn Diagram generation the online tool InteractiVenn was used (Heberle et al., 2015).

(c) Protein Annotation

Annotations for all detected proteins were generated by BlastP of each gene (*E. multilocularis* genome version WBPS 15, https://parasite.wormbase.org) against the Uniprot Database Eukarya (https://uniprot.org, 2022-10-01) with an e-value of 1e-5. Only top hits for each gene were included for further analyses. Top 25 bound proteins by both MEF and MEF-3 that did not get a hit by this BlastP analysis were analysed individually using the online tool of NCBI conserved domains (Lu et al., 2020). Functional grouping of these proteins was performed based on the information given in Uniprot.

(d) Gene Ontology (GO) enrichment analysis

For the analysis of GO terms two online tools were used: g:Profiler (Raudvere et al., 2019) and Enrichment Analysis in *Caenorhabditis elegans* (Angeles-Albores et al., 2016, 2018). Proteins bound exclusively by the effective drugs MEF and MEF-3 were subjected to this GO enrichment analysis using the respective *E. multilocularis* proteins as a query against the proteome of *C. elegans* (genome version WBPS 15, https://parasite.wormbase.org) with an e-value of 1e-5 to retrieve homologues in *C. elegans*. *C. elegans* is the only helminth species for which GO enrichment analysis is available to date. For further analyses and GO term enrichment was performed for the top hits of each protein in the three classic categories Cellular Component (CC), Biological Process (BP) and Molecular Function (MF), and the levels 3 and 4 are shown in the results. For each GO term the negative log(10) of the P value was plotted.

## 3 Results

### 3.1 MEF derivatives with activities against *E. multilocularis* metacestodes

**Fig. 3:**
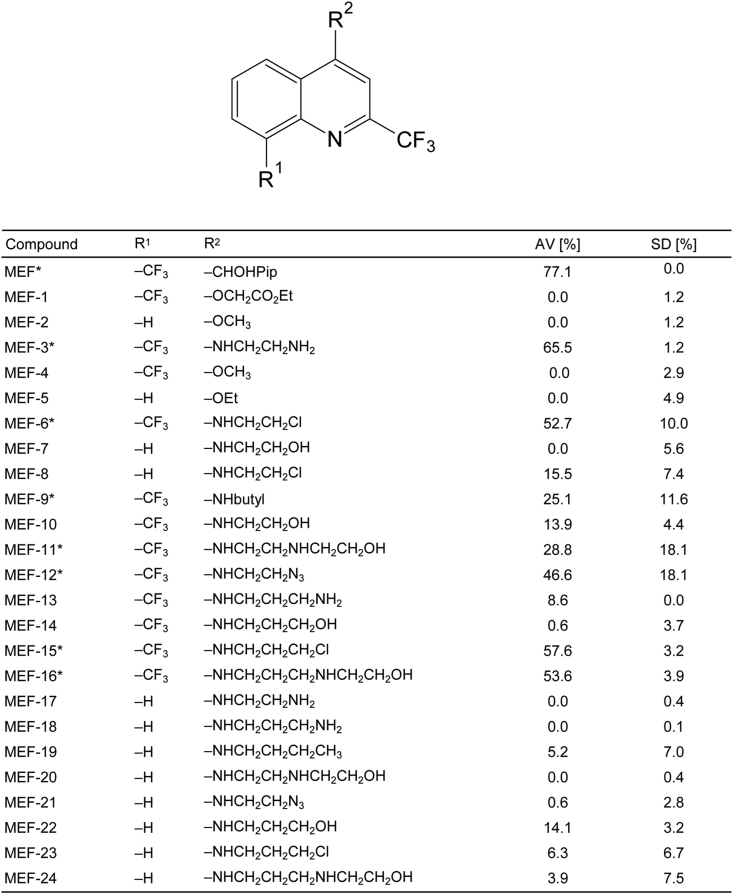
**Activity of MEF and derivatives against *E. multilocularis* metacestodes.** The compounds were tested at 40 μM against *E. multilocularis* metacestodes for 5 days under standard conditions (5% CO2, 21% O2, humid atmosphere) in biological triplicates. Average PGI values (AV) and standard deviations (SDs) are given in % of the positive control Tx-100 for each triplicate. Effective compounds (>20% PGI release) are indicated by *. For comparison, this table also includes the data published previously by Rufener et al. (2018) for MEF-1 – MEF-10.

In order to identify effective and ineffective MEF derivatives for subsequent analysis of affinoproteomes by DAC, an overview screening of the 14 MEF derivatives (MEF-11 – MEF-24) and MEF at 40 μM was performed to evaluate their activities against *E. multilocularis* metacestodes. Fig. 3 shows the PGI release relative to the positive control Tx-100 (for comparison, this table also includes the data published previously by Rufener et al. (2018) for MEF-1 – MEF-10). Four new compounds were effective (>20% of Tx-100 activity) in this first screen apart from MEF: MEF-11, MEF-12, MEF-15, and MEF-16. MEF-15 showed the highest activity compared to the other MEF derivatives, with a relative PGI activity of 57.6% (±3.2%) after 5 days of incubation. None of the MEF derivatives showed higher activities than MEF (77.1% ± 0.0%). The other MEF derivatives (MEF-13, MEF-14, MEF-17, MEF-18, MEF-19, MEF-20, MEF-21, MEF-22, MEF-23 and MEF-24) were ineffective after 5 days of incubation at 40 μM (Fig. 3).

**Fig. 4:**
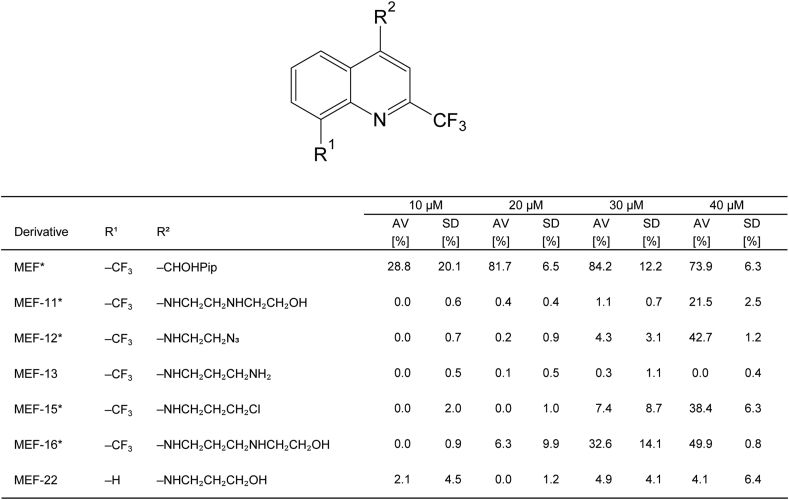
**Dose-dependent activity of MEF derivatives against *E. multilocularis* metacestodes.** Effective MEF derivatives from the screen at 40 μM (Fig. 2) and non-active MEF-13 and MEF-22 were re-assessed by PGI assay against *E. multilocularis* metacestodes at concentrations of 10, 20, 30 and 40 μM, for 5 days under standard conditions (5% CO2, 21% O2, humid atmosphere), and in biological triplicates. The activity of compounds was defined by the relative PGI-release compared to the positive control Tx-100. Average PGI values (AV) and standard deviations (SDs) are given for each triplicate. Effective compounds are indicated by *.

To confirm the MEF derivatives with activities of 20% or more in the first screen, they were tested in a PGI assay at concentrations of 10, 20, 30 and 40 μM. Fig. 4 confirms that none of the derivatives showed higher activities than MEF, which still exhibited an average relative PGI release of 81.7% (±6.5%) at 20 μM. MEF-16 showed the highest efficacy among the derivatives at 20 μM with an activity of 6.3% (±9.9%). MEF-13 and MEF-22 were also tested at lower concentrations as negative controls. They were again ineffective at all concentrations in these repeated tests.

### 3.2 DAC of MEF binding proteins

To identify potential protein targets of MEF in cell-free extracts of *E. multilocularis*, DAC was performed with the effective compounds MEF and MEF-3 and the ineffective MEF-13 and MEF-22. These compounds differ by the side chain in residue R^2^ and by the CF_3_ group in residue R^1^.

**Fig. 5:**
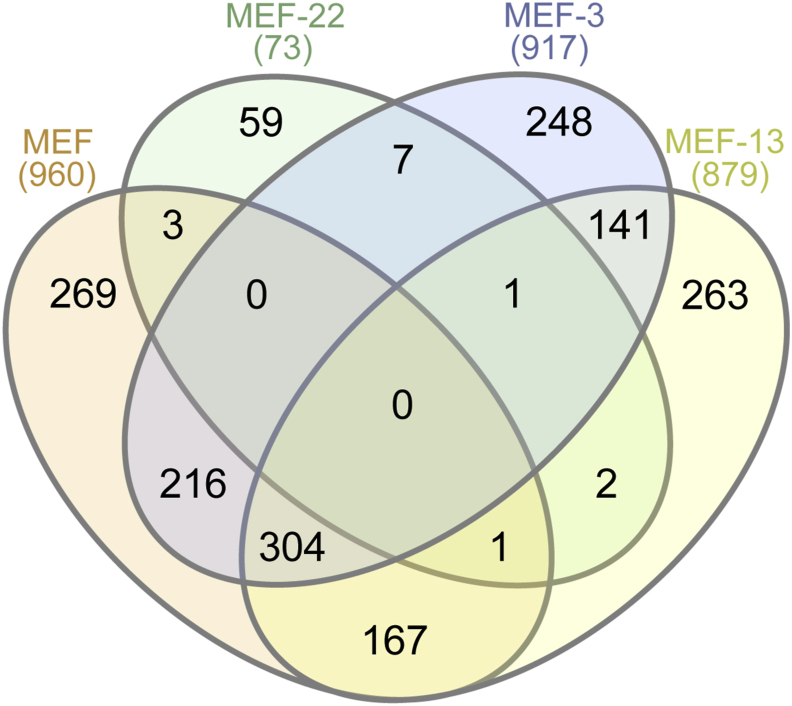
**Venn diagram of proteins from the *E. multilocularis* metacestode cell extract specifically binding to MEF or derivatives**. Numbers in parentheses indicate the total amount of proteins found for each drug-column. Numbers in the diagram indicate the amount of proteins for each section.

A total of 2′782 binding proteins were identified by this approach (Fig. 5, Supplementary Table S1). Contaminating proteins and proteins only identified by site were omitted from further analysis. Of the remaining 2′668 proteins (Supplementary Table S2), only those 1′681 binding proteins that were strictly detected in both duplicates per drug column, and not in the corresponding mock columns (Supplementary Table S3), were further analysed. Of all 1′681 proteins specific for MEF or derivatives, 960 proteins were detected in the MEF-coupled column, 917 proteins were detected in the MEF-3-coupled column, 879 proteins detected in the MEF-13-coupled column and 73 proteins were detected in the MEF-22-coupled column (Fig. 5). 269 proteins (16.00%) bound to MEF exclusively (Fig. 5, Supplementary Table S4) and 248 to MEF-3 exclusively (14.75%, Fig. 5, Supplementary Table S5). 263 (15.65%) proteins were detected only in the MEF-13 column. Ineffective MEF-22 bound 59 proteins (3.51%), of which three were bound to MEF as well. The 960 MEF-binding proteins included 167 proteins (9.93%) bound by ineffective MEF-13 and 216 proteins (12.85%) bound by effective MEF-3 as well (Supplementary Table S6, Fig. 5). These 216 proteins were regarded as the “MEF specific affinoproteome”.

**Fig. 6:**
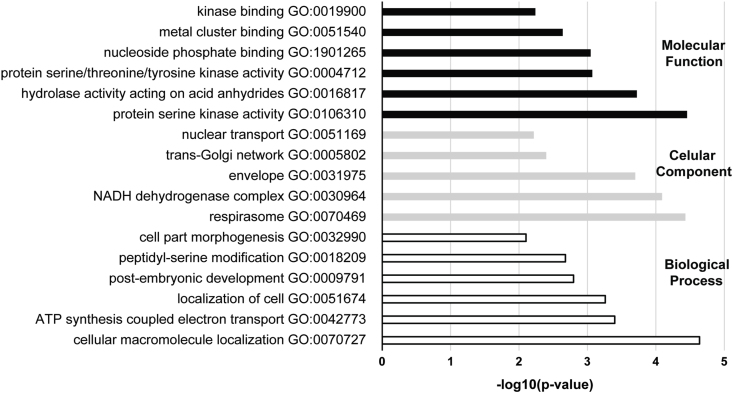
**Enriched GO terms detected by the binding proteins of the active compounds MEF and MEF-3.** The 216 proteins specifically bound by MEF and MEF-3 were subjected to GO term enrichment. The terms enriched in Molecular Function (black), Cellular Component (grey) and Biological Process (white) are shown with the respective negative log(10) of the P value. The GO terms here present are the ones detected in hierarchy levels 3 and 4.

GO enrichment analysis was performed for all the 216 “MEF specific” proteins (Fig. 6) in order to gain a better overview of these proteins’ functions. The significantly enriched GO terms in Biological Process were cellular macromolecule localization (GO:0070727), ATP synthesis coupled electron transport (GO:0042773), localization of cell (GO:0051674), post-embryonic development (GO:0009791), peptidyl-serine modification (GO:0018209), and cell part morphogenesis (GO:0032990). In Cellular Component the enriched GO terms were respirasome (GO:0070469), NADH dehydrogenase complex (GO:0030964), envelope (GO:0031975), trans-Golgi network (GO:0005802), and nuclear transport (GO:0051169). In Molecular Function the enriched GO terms were protein serine kinase activity (GO:0106310), hydrolase activity acting on acid anhydrides (GO:0016817), protein serine/threonine/tyrosine kinase activity (GO:0004712), nucleoside phosphate binding (GO:1901265), metal cluster binding (GO:0051540) kinase binding (GO:0019900).

Next, we had a closer look on the 25 most abundant proteins of the “MEF specific affinoproteome”. These proteins were sorted in function of their relative abundance calculated by the IBAQ algorithm (Table 1). The most abundant protein was the Universal Stress Protein PHOS32 (EmuJ_001076900.1). There were several other proteins involved in (i) stress response, protein folding and modification, as well as cell death (EmuJ_001050500.1, EmuJ_000025200.1, EmuJ_000569800.1, EmuJ_000190200.1, EmuJ_001060100.1, EmuJ_000796000.1, EmuJ_000881000.1). Highly abundant in this protein list were also proteins of (ii) mitochondrial energy metabolism and glucose homeostasis (EmuJ_001092300.1, EmuJ_000704000.1, EmuJ_000246800.1, EmuJ_000595000.1, EmuJ_001114700.1, EmuJ_000416200.1, EmuJ_000697900.1). Some of the proteins were involved in (iii) cellular transport and structure (EmuJ_000574700.1, EmuJ_001016100.1, EmuJ_000617100.1, EmuJ_000590100.1), or (iv) RNA/DNA binding (EmuJ_001062500.1, EmuJ_000652900.1, EmuJ_001104200.1). Three of these proteins could not be annotated, and therefore also no predictions on functions were made.

**Table 1:** **Top 25 proteins from the cell extract bound to both MEF and MEF-3 during affinity chro-matography and identified by nLC-MS/MS.** Out of the 216 proteins described as the “MEF specific affinoproteome”, the top 25 proteins were determined according to the highest iBAQ average from duplicates of MEF. The columns indicate the protein ID in WormBase ParaSite, the Protein ID and Protein Description of the best BlastP hit in Uniprot, or Conserved domain analysis where BlastP did not give any hits (indicated by *), as well as the iBAQ values for MEF and MEF-3.

Protein ID (Wormbase)	Protein ID (Uniprot)	Protein Description or Conserved domain analysis	iBAQ (MEF)	iBAQ (MEF-3)	Functional Grouping
EmuJ_001076900.1	Q8VYN9	Universal stress protein PHOS32	169750000	158330000	Cellular stress response, protein folding and modification, cell death
EmuJ_000474100.1	nd	* EF-hand calcium binding motif	110915000	8821300	NA
EmuJ_000574700.1	Q9R0Q3	Transmembrane emp24 domain-containing protein	79715000	18547000	Cellular transport and structure
EmuJ_000137050.1	nd	*Unknow function conserved domain with the conserved sequence motif LSWKL. Family that includes human protein C5orf43	78298500	24944000	NA
EmuJ_000474300.1	P13566	Calcium-binding protein	77138000	90818000	NA
EmuJ_001092300.1	Q9W141	Putative ATP synthase subunit f, mitochondrial	70382500	21309000	Mitochondrial energy metabolism and glucose homeostasis
EmuJ_001016100.1	O77735	Secretory carrier-associated membrane protein 1	64028500	5038245	Cellular transport and structure
EmuJ_001050500.1	Q7PXE2	Ubiquitin-fold modifier 1	62325500	165335000	Cellular stress response, protein folding and modification, cell death
EmuJ_000704000.1	B4F6K2	Hydroxyacylglutathione hydrolase, mitochondrial	60756000	116217500	Mitochondrial energy metabolism and glucose homeostasis
EmuJ_000246800.1	Q5XIU9	Membrane-associated progesterone receptor component 2	55423000	82396500	Mitochondrial energy metabolism and glucose homeostasis
EmuJ_000025200.1	O75340	Programmed cell death protein 6	54433000	2770950000	Cellular stress response, protein folding and modification, cell death
EmuJ_000569800.1	Q66HA6	ADP-ribosylation factor-like protein 8B	50569000	60730000	Cellular stress response, protein folding and modification, cell death
EmuJ_001062500.1	Q9CW03	Structural maintenance of chromosomes protein 3	45809000	81300500	Nucleic acid binding
EmuJ_000595000.1	Q9UBX3	Mitochondrial dicarboxylate carrier	42766500	7365950	Mitochondrial energy metabolism and glucose homeostasis
EmuJ_000652900.1	Q62280	Protein SSXT	38922500	23638000	Nucleic acid binding
EmuJ_000617100.1	Q9W4P5	V-type proton ATPase subunit d 1	38462000	7309100	Cellular transport and structure
EmuJ_000190200.1	Q5RA95	Protein NDRG3	36578000	21148500	Cellular stress response, protein folding and modification, cell death
EmuJ_001114700.1	Q9D6J6	NADH dehydrogenase [ubiquinone] flavoprotein 2, mitochondrial	35148000	98142500	Mitochondrial energy metabolism and glucose homeostasis
EmuJ_001104200.1	Q01617	Protein couch potato	32180000	13055550	Nucleic acid binding
EmuJ_001060100.1	Q5ZKC9	14-3-3 protein zeta	31950500	140195000	Cellular stress response, protein folding and modification, cell death
EmuJ_000590100.1	Q24117	Dynein light chain 1, cytoplasmic	29960500	23792675	Cellular transport and structure
EmuJ_000416200.1	P21912	Succinate dehydrogenase [ubiquinone] iron-sulfur subunit, mitochondrial	28923500	35678000	Mitochondrial energy metabolism and glucose homeostasis
EmuJ_000697900.1	Q9CQJ8	NADH dehydrogenase [ubiquinone] 1 beta subcomplex subunit 9	28034500	8235050	Mitochondrial energy metabolism and glucose homeostasis
EmuJ_000796000.1	E2AXC7	Lys-63-specific deubiquitinase BRCC36	27714000	41773500	Cellular stress response, protein folding and modification, cell death
EmuJ_000881000.1	P36872	Protein phosphatase PP2A 55 kDa regulatory subunit	27286000	69148000	Cellular stress response, protein folding and modification, cell death

## 4 Discussion

Infection with *E. multilocularis* larvae causes the disease AE with infiltrative growth in the liver (Stojkovic and Junghanss, 2013). The high fatality rate in untreated patients makes AE one of the most dangerous food-borne parasitoses in the world (Lymbery et al., 2017). New chemotherapeutic treatment options are urgently needed. Drug repurposing is a strategy that might efficiently aid in the identification of alternative drug treatments for AE, as for other neglected diseases (Panic et al., 2014; Lundström-Stadelmann et al., 2020). One of the most important heterocyclic compounds in the field of medicinal and organic chemistry is quinoline, which occurs in the backbone of molecules with great biological importance of natural (Musiol et al., 2017; Nainwal et al., 2019) or synthetic origin (Narwal et al., 2017; Sharma et al., 2017; Shang et al., 2018), such as quinine, camptothecin or skimminanine (Musiol et al., 2017; Nainwal et al., 2019). These bioactive quinoline-based compounds were the precursors of several derivatives that are now used clinically and contribute greatly to the control of serious diseases worldwide (Narwal et al., 2017; Sharma et al., 2017; Shang et al., 2018; Kucharski et al., 2022). In addition, several other compounds with quinoline nuclei fused or unfused to other heterocyclic compounds have been synthesized recently, exhibiting a wide range of biological properties *in vitro* and *in vivo*, such as antimalarials (Leven et al., 2019), antifungals (Shruthi et al., 2019), anticancer agents (Hamdy et al., 2019), anticonvulsants (Kumar and Abdullah, 2019), antihypertensives (Vellalacheruvu et al., 2017), anti-inflammatories (Deaton et al., 2019), antidepressants (Galambos et al., 2017), and antivirals (Kos et al., 2019).

For MEF, which has a quinoline nucleus substituted by two trifluoromethyl groups, activity against *E. multilocularis* metacestodes was demonstrated in recent years (Küster et al., 2011, 2015; Lundström-Stadelmann et al., 2020). Even though the drug is active *in vitro* at relatively high concentrations, the same holds true for the current drug in use, albendazole. In the murine AE model, mefloquine is the only drug that to date reaches comparable parasite reduction as albendazole. Building on previous studies that evaluated the importance of specific chemical groups for MEF activity, new analogues were evaluated in the present study to determine likely mechanisms of action for MEF and its derivatives. Our *in vitro* testing of 14 new derivatives of MEF against *E. multilocularis* metacestodes clearly showed that none of the 14 MEF derivatives showed higher activity compared to MEF in the PGI assay against *E. multilocularis* metacestodes. This is in contrast to a previous study where MEF-21 and MEF-23 showed activity against resistant *Mycobacterium tuberculosis* strains without causing toxicity to human macrophage cells (da Silva et al., 2021). However, four compounds (MEF-11, MEF-12, MEF-15 and MEF-16) caused limited physical damage to *E. multilocularis* metacestodes.

SAR is a very useful to follow structural modifications of a compound to optimize its property or activity (Guha, 2013). In order to find new effective derivatives of MEF, which ideally would be less toxic than MEF, the structures of MEF derivatives were compared in a SAR study. MEF-derivatives 17–24 were found to be ineffective against metacestodes. Their common feature was the lack of a trifluoromethyl group at position 8 (R^1^) of the quinoline core structure. These findings are consistent with previous results obtained with MEF derivatives (MEF-1 – MEF-10) tested *in vitro* against *E. multilocularis* metacestodes (Rufener et al., 2018). In contrast, MEF-13 and MEF-14, which contained a trifluoromethyl group at R^1^ and a secondary amine in R^2^, showed no activity against metacestodes. Similar studies by Dassonville-Klimpt et al. (2011) and Barbosa-Lima et al. (2017) showed that derivatives containing trifluoromethyl groups were effective against *Plasmodium* spp. and secondary amine side chains were effective against the Zika virus. It appears that the inclusion of a chlorine atom in the side chain at position 4 (R^2^) contributes to the increased activity for MEF-7 *versus* MEF-8, MEF-14 *versus* MEF-15, MEF-10 *versus* MEF-6, MEF-17 *versus* MEF-8 and MEF-22 *versus* MEF-23, with the exception of MEF-3 *versus* MEF-6. Small groups located at position 4 of the ring (R^2^) provided inactive derivatives, for example MEF-2, MEF-4 and MEF-5. Inclusion of a methylene group in the side chain at the R^2^ of MEF-3 resulted in the less active derivative MEF-13. The same was also observed forMEF-10 that lost its activity with an additional methylene group in MEF-14. On the other hand, the insertion of a methylene group in the side chain of MEF-6 resulted in a more active derivative MEF-15, and the same was observed in MEF-11 in relation to MEF-16. MEF-15, which contained three methylene groups, was effective and differed from MEF-13 and MEF-14 in that it contained a chlorine at the end instead of a primary amine or hydroxyl group. The fact that most of the derivatives such as MEF-11, MEF-12, MEF-16 and the first-generation derivatives from Rufener et al. (2018), contained good nucleophiles at the end of the side chain suggests that nucleophiles could play a role in the activity, but could be influenced by the length of the side chain, as in the case with MEF-13 and MEF-14.

Concerning the length of the side chain, another observation was made with MEF-15 and MEF-16. It can be assumed that MEF-16, the most promising second-generation effective derivative against *E. multilocularis* metacestodes, showed activity due to the similar functional groups with a total of 8 atoms in the long R^2^ residue as in the 2-piperidylmethanol group of MEF. Therefore, one idea of a possible interaction of MEF-16 with the target could be in mimicking the 2-piperidylmethanol structure of MEF. An interaction of the 2-piperidylmethanol of MEF with amino acids in a binding pocket was demonstrated in a previous study where MEF bound to acyl-CoA-binding proteins of *Plasmodium falciparum* (Kumar et al., 2021). Further research with new derivatives is needed to broaden the SAR study of MEF.

The phenotypical screening of MEF derivatives has provided us with one effective (MEF-3) and two ineffective derivatives MEF-13 and MEF-22 with structural similarity to MEF. Consequently, DAC using these compounds was performed in order to identify *E. multilocularis* proteins specifically binding to MEF or effective MEF derivatives resulting in the identification of 1′681 proteins. A potential downside of affinity chromatography upon drug-coupling to a solid support lays in that by coupling the potential target-binding affinity can be changed. However, the same basic method of coupling of MEF for affinity chromatography was applied before (Manneck et al., 2012; Küster et al., 2015). Moreover, the low number of exclusively detected proteins bound by MEF-22 (3.51%) and the corresponding high number of exclusively bound proteins by MEF (16.00%) and by MEF-3 (14.75%) were consistent with the expectation regarding protein binding in relation to activity. What was unexpected was the comparatively equal number of proteins bound exclusively to the ineffective MEF-13 (15.65%). Despite the biological inactivity of MEF-13 towards *E. multilocularis* metacestodes, the protein binding ability could be explained by the fact that the trifluoromethyl group (R^1^ residue) contained at position 8, which is not present in the structure of MEF-22, interacts non-specifically with many proteins.

As shown before (Müller et al., 2022a), DAC is a useful tool to identify affinoproteomes specific for effective compounds. In the present study, the use of biologically ineffective analogues of MEF with high similarity to the effective compounds allows to remove non-specifically bound proteins from the affinoproteome of effective MEF-analogues. However, with 216 proteins this specific affinoproteome is still very large.

Since most of these 216 binding proteins are only detectable in small amounts, it is safe to focus on the 25 most abundant proteins bound by both effective compounds MEF and MEF-3. These “Top 25” include proteins involved in pathways of (i) stress response and protein modification and folding, (ii) energy metabolism, (iii) cellular transport and structure, and (iv) nucleic acid binding. In order to not limit the functional grouping to the top 25 listed proteins, GO term enrichment was performed on the entire dataset of MEF and MEF-3 binding proteins. GO term enrichment is not yet readily available for neglected parasites like cestodes, but could be adapted from publicly available enrichment tools for the helminth *C. elegans*. By this approach highly significant GO hits reflected a very similar grouping as the top 25 proteins. Highly interestingly, many proteins were involved in energy metabolism (GO terms: ATP synthesis coupled electron transport, respirasome, NADH dehydrogenase complex). This is in line with previous studies that found the energy metabolism of Schistosomes, *Streptococcus* and mammalian cells to be affected by MEF (Martín-Galiano et al., 2002; Manneck et al., 2012; Li et al., 2017; McCann et al., 2018). An important consideration is that binding to a drug does not necessarily lead to functional inhibition. Therefore, follow-up experiments will be needed to prove a functional inhibition of the energy metabolism by MEF.

The binding of proteins involved in cellular transport and structure is also in line with previous observations made in the *in vivo* study by Küster et al. (2015), where MEF treatment of *E. multilocularis* metacestodes led to destructive effect on microtriches, which are essential for microtubule-dependent vesicle transport into the laminated layer (Küster et al., 2011).

Stress response and protein modification and nucleic acid binding were further terms the top 25 proteins and GO enrichment of all MEF and MEF-3 specific binding proteins covered. Whereas MEF induces reactive oxygen species and cell death in target cells, it has, to the best of our knowledge, not been shown that specific cell death proteins, proteins of protein modification or folding, nor stress response or nucleic acid, are bound by MEF (Ghosh et al., 2021). They might be highly upregulated in response to MEF treatment, and thereby non-specifically bound to MEF, or MEF could bind central stress response proteins actively and thereby further weaken the parasite. Targeted experiments in the future will show, if MEF also directly affects stress response pathways in the parasite.

In the study by Küster et al. (2015), where a limited and biased proteomic approach based on in-gel-digestion of protein bands was used to identify potential drug interaction partners of MEF, ferritin and cystatin were identified. In other parasites, MEF probably inhibits β-haematin formation during hemoglobin degradation in Plasmodia (Egan et al., 1994; Corrêa Soares et al., 2009) or, respectively, glycolytic enolase in Schistosomes (Manneck et al., 2012). Therefore, it was postulated that MEF might bind metalloproteins like ferritin, haematin or enolase via its strongly electronegative trifluoromethyl group that could interact with metals bound by these metalloproteins. This hypothesis is further backed up by the GO term metal cluster binding that was identified highly enriched amongst the MEF and MEF-3 binding proteins. Further, in our previous SAR study with MEF derivatives it was shown that 4-aminoquinoline derivatives with a trifluoromethyl group in position eight were necessary for *in vitro* activity against metacestodes (Rufener et al., 2018). In the current more comprehensive DAC study with 14 additional MEF derivatives, ferritin was not ranked within the top 25 binding proteins, but was still included in the repertoire of proteins bound by MEF exclusively. One explanation for this could be the different elution procedure in the previous study, where elution was performed with MEF to attract bound proteins (i.e. specific elution). This may have resulted in a bias towards metalloprotein abundance in the eluates and would also explain why no cystatin was detected in the present study as to compared to the previously performed study (Küster et al., 2015). Further, we could show that ferritin was only bound by MEF and not shared by biologically active MEF-3 and other MEF derivatives. This might explain why MEF showed superior activity against *E. multilocularis* metacestodes, and might be an additional confirmation of ferritin being a central protein in the activity of MEF against this tapeworm. Future studies will have to address the direct binding of the intracellular iron-storage protein ferritin by MEF.

Also past studies outside the field of *Echinococcus* have shown that MEF targets multiple pathways: In *Plasmodium*, it blocks protein synthesis by targeting the cytosolic 80S ribosome, it affects lipid-binding proteins, it inactivates the enzyme metacapsase-1, and it inhibits heme polymerization. Further, MEF was shown to act also on cancer and bacterial cells, and in humans, it was shown to induce hepatic, gastrointestinal, as well as neuropsychiatric side effects. These are mediated as well through multiple pathways, ranging from membrane disorganization, imbalance of small molecule transmembrane transport, deregulated lipid metabolism, destabilization of organelle homeostasis, interruption of signaling pathways, including various described protein targets, yet still the detailed causal mechanisms remain unclear (Ghosh et al., 2021).

## 5 Conclusions

The aim of this study was to assess the effecicacy of MEF derivatives against *E. multilocularis* metacestodes and evaluate their interaction with its proteome to better understand the mode of action of MEF. Based on the drug efficacy assays it can be concluded that none of the derivatives were more effective than MEF against *E. multilocularis* metacestodes. MEF-16 was one of the most potent among the derivatives. Its structure suggests that, in addition to the trifluoromethyl group, the length of the second residue with similar functional groups to the 2-piperidylmethanol in MEF is important. Using DAC followed by nLC-MS/MS, proteins binding to MEF and MEF-3, but not to ineffective MEF derivatives, were identified. The majority of these binding proteins – thus potential targets – are involved in energy metabolism and cellular transport and structure, and possibly other pathways as well thereby explaining the broad spectrum of MEF against various pathogens and its side effects on hosts.

## Appendix A Supplementary data

Supplementary Table S1 Overview of *E. multilocularis* proteins bound to MEF and MEF derivatives. Identified total proteins (n = 2′782) in all samples (proteins bound to MEF, MEF-3, MEF-13, MEF-22). *LFQ (Label-Free-Quantification)*: values produced by MaxQuant's own algorithm. *Top3 (sum of the three most intense peptide intensities)*: as an alternative to LFQ. *iBAQ (Intensity Based Absolute Quantification)*: values are the intensities divided by the number of tryptic peptides. *Normalisation*: peptides are first normalised (variance stabilisation) before being summed up for Top3. *Razor peptides*: Shared common peptides between protein groups, which are unambiguously identified via unique peptides. The razor peptide will be assigned to the protein group with the largest number of total peptide identified. *Razor + unique peptides*: The total number of razor and unique peptides associated with the protein group (i.e. these peptides are shared with another protein group). *Sequence coverage [%]*: Percentage of the sequence that is covered by the identified peptides of the best protein sequence contained in the group. *Q-value*: ratio of reverse to forward protein groups. *Score*: Protein score derived from peptide posterior error probabilities (PEP). *Intensity*: intensities are the sums of all individual peptide intensities belonging to a particular protein group. Unique and razor peptide intensities are used as default. *iBAQ peptides*: tryptic peptides. *MS/MS count*: Peptide spectrum matches. *Only identified by site*: Particular protein group identified only by a modification site. *Potential contaminant*: commonly occurring contaminant, which is removed from further data analysis. Supplementary Table S1

Supplementary Table S2 Overview of *E. multilocularis* proteins bound to MEF and MEF derivatives (n = 2′668). Identified total proteins without contaminants and proteins only identified by site removed in all samples (MEF, MEF-3, MEF-13, MEF-22). Supplementary Table S2

Supplementary Table S3 Overview of *E. multilocularis* proteins bound to MEF or any of the derivatives (n = 1′681). These proteins are detected in both replica for each drug column, but not in the corresponding mock columns (Subset of Supplementary Table S2). Supplementary Table S3

Supplementary Table S4 Overview of *E. multilocularis* proteins bound only to MEF (n = 269). Subset of Supplementary Table S3 showing only proteins exclusively bound to MEF and not MEF-3, MEF-13 or MEF-22. Supplementary Table S4

Supplementary Table S5 Overview of *E. multilocularis* proteins bound only to MEF-3 (n = 248). Subset of Supplementary Table S3 showing only proteins exclusively bound to MEF-3 and not MEF, MEF-13 or MEF-22. Supplementary Table S5

Supplementary Table S6 Overview of shared *E. multilocularis* proteins bound to MEF and MEF-3 (n = 216). Subset of Supplementary Table S3 showing only proteins exclusively bound to MEF and MEF-3 and not MEF-13 or MEF-22. Also termed the “MEF specific affinoproteome” in this study. Supplementary Table S6
